# Estimates of hospitalisations and deaths in patients with COVID-19 associated with undiagnosed diabetes during the first phase of the pandemic in eight low-income and middle-income countries: a modelling study

**DOI:** 10.1016/j.eclinm.2024.102492

**Published:** 2024-03-05

**Authors:** Amit Summan, Arindam Nandi, Brian Wahl, Sergio Carmona, Stefano Ongarello, Beatrice Vetter, Ramanan Laxminarayan

**Affiliations:** a One Health Trust, Washington, DC, USA; b The Population Council, NY, USA; c Department of International Health, Johns Hopkins Bloomberg School of Public Health, Baltimore, USA; d FIND, Geneva, Switzerland; e One Health Trust, New Delhi, India; f High Meadows Environmental Institute, Princeton University, Princeton, USA

**Keywords:** COVID-19, Diabetes, Undiagnosed diabetes, Non-communicable diseases

## Abstract

**Background:**

Patients with COVID-19 that had diagnosed chronic diseases — including diabetes — may experience higher rates of hospitalisation and mortality relative to the general population. However, the burden of undiagnosed co-morbidities during the pandemic has not been adequately studied.

**Methods:**

We developed a model to estimate the hospitalisation and mortality burden of patients with COVID-19 that had undiagnosed type 1 and type 2 diabetes (UD). The retrospective analytical modelling framework was informed by country-level demographic, epidemiological and COVID-19 data and parameters. Eight low-and middle-income countries (LMICs) were studied: Brazil, China, India, Indonesia, Mexico, Nigeria, Pakistan, and South Africa. The modelling period consisted of the first phase of the pandemic — starting from the date when a country identified its first COVID case to the date when the country reached 1% coverage with one dose of a COVID-19 vaccine. The end date ranged from Jan 20, 2021 for China to June 2, 2021 for Nigeria. Additionally, we estimated the change in burden under a scenario in which all individuals with UD had been diagnosed prior to the pandemic.

**Findings:**

Based on our modelling estimates, across the eight countries, 6.7 (95% uncertainty interval: 3.4–11.3) million COVID-19 hospitalised patients had UD of which 1.9 (0.9–3.4) million died. These represented 21.1% (13.4%–30.1%) of all COVID-19 hospitalisations and 30.5% (14.3%–55.5%) of all COVID-19 deaths in these countries. Based on modelling estimates, if these populations had been diagnosed for diabetes prior to the COVID-19 pandemic, 1.7% (−3.0% to 5.9%) of COVID-19 hospitalisations and 5.0% (−0.9% to 14.1%) of COVID-19 deaths could have been prevented, and 1.8 (−0.3 to 5.0) million quality-adjusted life years gained.

**Interpretation:**

Our findings suggest that undiagnosed diabetes contributed substantially to COVID-19 hospitalisations and deaths in many LMICs.

**Funding:**

This work was supported, in part, by the 10.13039/100000865Bill & Melinda Gates Foundation [INV-029062] and FIND.

**Research in context**

**Evidence before this study**

We searched PubMed and Google Scholar databases on May 29, 2023, using search terms that included variations and combinations of the phrases “undiagnosed”, “diabetes”, “newly”, “burden”, “hospitalisations”, “deaths”, and “COVID-19” without any date, language, or publication type restrictions. While previous literature has found COVID-19 infected populations with co-morbidities — such as hypertension, obesity, and diabetes — experienced more severe outcomes than healthy populations and estimated the proportion of COVID-19 patients with these underlying diseases who experienced hospitalisation and death, they have focused on diagnosed diseases. There is a lack of estimates of the burden of undiagnosed NCDs, including diabetes, during the COVID-19 pandemic. Additionally, the relative risks of severe outcomes for undiagnosed diabetes populations are relatively unknown. We identified a meta-analysis published in November 2020 which included three studies that report the prevalence of previously diagnosed vs. previously undiagnosed diabetes in patients with COVID-19.

**Added value of this study**

We estimated the prevalence of undiagnosed diabetes in COVID-19 hospitalised populations and mortality within these hospitalised populations in eight low-income and middle-income countries. An understanding of the risk factors driving severe COVID-19 outcomes can drive targeted investments in prevention and control efforts to reduce health system burdens during future pandemic waves.

**Implications of all the available evidence**

The growing burden of non-communicable diseases (NCDs) necessitates increased investments in prevention and diagnostics. NCDs can severely increase the health burden caused by new and emerging infectious diseases.

## Introduction

Non-communicable diseases (NCDs) contribute to a large health burden in low-income and middle-income countries (LMICs). In 2019, NCDs were responsible for 719 million disability-adjusted life years (DALYs) lost in LMICs according to the Global Burden of Disease study.^1^ The importance of this large NCD burden has been underscored during the novel coronavirus SARS-COV-2 (COVID-19) pandemic.^2^

A substantial proportion of the burden of hospitalisations and mortality caused by COVID-19 has been attributed to NCDs, particularly hypertension, diabetes, and chronic obstructive pulmonary disease (COPD).^2^ Moreover, cancer, cardiovascular diseases, diabetes, and COPD were observed to be worsened by direct damage to major organs caused by COVID-19.^3^ A meta-analysis found that diabetes was the third most common co-morbidity associated with higher COVID-19 severity and mortality after hypertension and obesity.^2^ However, accounting of diabetes in patients with COVID-19 has focused on populations with known diabetes, potentially excluding the 45%^4^ of individuals with diabetes globally that are not aware of their status.

While meta-analyses have documented country-level prevalence of diabetes for COVID-19 infected populations with different clinical outcomes,^2^^,^^5^ there is a dearth of information regarding the outcomes of those previously undiagnosed for these conditions, particularly in LMIC settings. One meta-analysis estimated population attributable fractions (PAFs) of diabetes across countries, by reviewing studies that documented the prevalence of diabetes in patients with COVID-19.^6^ For these PAFs to include previously undiagnosed diabetes requires an implicit assumption that the reviewed studies involved diagnosis and testing of previously undiagnosed patients. However, patient co-morbidities were self-reported in several health centre and surveillance studies.^7^, ^8^, ^9^ Considering that many studies were conducted in settings where health systems were overwhelmed with patients with COVID-19,^10^^,^^11^ it is likely that rates of diagnostic testing for diabetes were low. There are a few health centre studies that focus specially on previously undiagnosed diabetes in patients with COVID-19. One meta-analysis reviewed three studies — two from China and one from the United States — that identified prevalence of previously undiagnosed diabetes in hospitalised patients with COVID-19.^12^ They noted additional studies from Italy and the United States where the difference between new-onset diabetes and previously undiagnosed diabetes could not be made. Another meta-analysis^13^ focused on new-onset diabetes and identified studies in Europe, China, and the USA — where this literature remains concentrated — but these studies did not distinguish between new-onset diabetes and previously undiagnosed diabetes. The overall burden of key undiagnosed NCDs in patients with COVID-19 who were hospitalised or died, and the potential value of prevention, early diagnosis, and treatment of these NCDs prior to the pandemic, remains largely unquantified.

It is valuable to understand the burden of undiagnosed diabetes during COVID-19 for several reasons. First, patients with COVID-19 that have undiagnosed, untreated, and uncontrolled diabetes^14^ may have higher risks of severe hospitalisation and mortality relative to those with prior diagnosis or treatment. Reducing hospitalisations alleviates the burden on LMIC health systems, which have been significantly challenged during the pandemic. Second, appropriate accounting of the risk factors driving COVID-19 mortality will aid in determining the appropriate policy response to potential future COVID-19 waves and other pandemics. Lastly, estimation of the burden caused by lack of diagnosis in LMICs will encourage much-needed investment in detection, treatment, and control of diabetes in the future. In this paper, we assessed the burden of undiagnosed diabetes during the first phase of the COVID-19 pandemic for eight populous LMICs.

## Methods

### Overview

The prevalence of undiagnosed diabetes (both type 1 and 2) in COVID-19 hospitalised patients older than 20 years of age was estimated in eight countries — Brazil, China, India, Indonesia, Mexico, Nigeria, Pakistan, and South Africa — with a retrospective analytical model. These countries were chosen given their relatively large population sizes, together representing 49% of the global population, and their geographical diversity. Also, the potential avertable burden due to diagnosis was estimated under an intervention scenario where undiagnosed populations were modelled as being diagnosed prior to the pandemic. We focused on the first phase of the pandemic for each country, which was defined as the period between the country identifying its first COVID 19 case to when the country’s population reached a 1% vaccination rate with one dose of a COVID-19 vaccine. We opted to not include the post-vaccination period given the paucity of data on the effectiveness of different COVID-19 vaccines against hospitalisations and death among those with untreated diabetes. According to our definition, the first phase of the pandemic varied from an end date of Jan 20, 2021, for China to June 2, 2021, for Nigeria. The outcomes studied were hospitalisations, deaths, and quality-adjusted life years (QALYs).

To estimate the burden of undiagnosed diabetes, we required the adjusted relative risk of hospitalisation and mortality for patients with COVID-19 that had undiagnosed vs. diagnosed diabetes. We identified a November 2020 meta-analysis that describes the prevalence of previously undiagnosed and newly diagnosed diabetes in COVID-19 hospitalised patients from three studies.^12^ The meta-analysis defined previously undiagnosed diabetes as follows: no previous history of diabetes and fasting plasma glucose ≥7.0 mmol/L or random blood glucose ≥11.1 mmol/L and HbA1c ≥ 6.5% or HbA1c ≥ 6.5% only. Only one of the included studies^14^ reported mortality outcomes. This study^14^ provided the adjusted relative risk of mortality in patients with COVID-19 that had previously undiagnosed diabetes vs. diagnosed diabetes controlling for age, sex, smoking, systolic blood pressure, total cholesterol, use of antihypertensive drugs or lipid-lowering agents, admission to intensive care unit, and use of invasive mechanical ventilation, and was used in our analysis. The hazard ratio of those with known diabetes relative to normal glucose was 6.01 and those with previously undiagnosed diabetes relative to normal glucose was 7.21.^14^ Based on these relative hazard ratios, we estimated the relative risk of mortality of those with previously undiagnosed to previously diagnosed as 1.19. This relative risk is comparable to other diseases; for example, one cohort study estimated that the relative risks for patients with uncontrolled vs. controlled diabetes for all-cause mortality, congenital heart disease hospitalisation, and stroke hospitalisation were 1.29, 1.38, and 1.05, respectively.^15^ Another study estimated the relative risk of mortality to be 2.5 and 2.7 for those with diagnosed diabetes vs. undiagnosed diabetes respectively, as compared to normoglycemic individuals.^16^

### Statistical analysis

#### Hospitalisations

Hospitalisation rates for patients with COVID-19 are unavailable for many countries, particularly LMICs, due to lack of tracking data. However, confirmed deaths have been tracked relatively better. Therefore, we estimated the number of hospitalised patients for each country as follows:

$${Hos}_{c}=\frac{1}{{CFR}_{R}}*D_{c}$$

Where ${CFR}_{R}$ is the region-specific case fatality rate of hospitalised patients and $D_{C}$ is the estimated number of COVID-19 deaths in country c. To estimate the CFR by region, we took a global CFR of 20% for COVID-19 hospitalised patients from a meta-analysis of 38, 000 patients in 77 studies^5^ and adjusted it for local epidemiological and demographic characteristics. The adjustment was done based on a study^17^ that estimated the proportion of the population at-risk of severe COVID-19 in each geographical region, based on rates of co-morbidity and COVID-19 hospitalisation, and age and sex distribution. Both globally and in Asia, an estimated 4.5% of the population was at risk of severe COVID. We scaled the CFR for African and Latin American regions based on their at-risk population relative to Asia — this resulted in a 31% and 9% scaling down of the CFR, respectively. Confirmed deaths came from the Our World in Data database^18^ and were scaled up according to scaling factors found in a study^19^ that compared confirmed deaths to total actual deaths using excess mortality data. Scaling factors are shown in Table 1.

**Table 1:** Country demographic and epidemiological characteristics.

Country	Date reached 1% vaccination	Reported COVID-19 deaths	Population	Diabetes prevalence	Proportion of diabetes population with undiagnosed diabetes	Death adjustment scaling factor	Years of life lost per COVID death in diabetes patients
Brazil	2/1/21	468,086	214,317,000	0.09	0.32	1.28	7.14
China	1/20/21	4635	1,412,000,000	0.11	0.52	3.71	9.14
India	2/26/21	513,724	1,380,000,000	0.10	0.53	8.33	10.63
Indonesia	3/2/21	36,518	271,350,000	0.11	0.74	5.11	4.85
Mexico	2/18/21	178,108	126,014,024	0.17	0.48	1.91	10.92
Nigeria	6/2/21	2099	211,411,000	0.04	0.53	36.37	11.60
Pakistan	5/4/21	18,310	225,220,000	0.31	0.27	22.99	11.41
South Africa	5/20/21	55,568	60,142,978	0.11	0.46	3.31	12.33
Source	Our World in Data^18^		United Nations^20^	International Diabetes Federation^4^		Wang et al.^19^	Authors’ assumption based on Pifarre et al.,^21^ United Nations,^20^ Franco et al.^22^

Note: The number of deaths are provided for on the date for which the country reached 1% vaccination. Death adjustment scaling factor is based on estimates of under-reporting of official COVID-19 deaths based on excess mortality data.

Then the proportion of the total population with known diabetes that was hospitalised for COVID-19 was estimated using the following equation:

$${hr}_{DD,c}=\frac{{Hos}_{c}\;*p_{DD,c}^{h}\;}{{{POP}_{c}*p}_{D,c}*\left(1-p_{UD,c}\right)}$$

Where ${hr}_{DD,c}$ is the proportion of the diagnosed diabetes (DD) population (aware of their diabetes status) which was hospitalised for COVID-19 in country *c*, $p_{DD,c}^{h}$ is the proportion of COVID-19 hospitalised patients which have DD in country *c*, ${POP}_{c}$ is the total population over age 20, $p_{D,c}$ is the proportion of population with diabetes, and $p_{UD,c}$ is the proportion of the population with undiagnosed diabetes (UD). Estimates of the proportion of hospitalised patients with COVID-19 that had previously DD were taken from country-specific studies and are presented in Table 2. The prevalence of known and unknown diabetes in the adult population by country was taken from the World Diabetes Atlas.^4^

**Table 2:** Prevalence of diabetes among COVID-19 hospitalised patients.

Country	Prevalence^a^	Study period	Study setting	Sample size	Source
Brazil	0.161^b^	Mar 10–Nov 13, 2020	Santa Catarina Hospital, São Paulo	1170	Tamura et al.^23^
China	0.079 (0.066–0.092)	Based on meta-analysis of six China based studies.		1714	Emami et al.^24^
India	0.177 (0.122–0.251)	Based on meta-analysis of 34 India based studies.		23,034	Jindal et al.^25^
Indonesia	0.120^b^	Mar 2–Jul 31, 2020	55 hospitals in Jakarta	4625	Surendra et al.^9^
Mexico	0.329^b^	Mar 1–Nov 13, 2020	National level data from health centres covered by Mexican Social Security Institute	121,225	Peña et al.^8^
Nigeria	0.166 (0.112–0.228)	Based on meta-analysis of 12 studies based in African region		10,756	Li et al.^6^
Pakistan	0.294^b^	Feb–Aug 2020	Four hospitals in Islamabad-Rawalpindi	1812	Akhtar et al.^26^
South Africa	0.166 (0.112–0.228)	Based on meta-analysis of 12 studies based in African region		10,756	Li et al.^6^

a Mean prevalence and 95% confidence interval provided in parentheses.

b Confidence interval not reported.

A key assumption of our model is that during the first phase of the pandemic, there was limited to no new diagnosis of diabetes in patients with COVID-19 in health centre settings. This is based on our review of studies that examined diabetes prevalence in COVID-19 hospitalised patients (Table 2). In a meta-analysis in the context of India,^25^ the largest cited study noted that all co-morbidities were self-reported.^7^ In the second largest study, it was unclear if co-morbidities were self-reported or tested for, but the authors noted that patient body weights were not measured due to staff time constraints, suggesting an overburdened health system overall.^11^ Diabetes prevalence in hospitalised patients in Mexico came from the Mexico Social Security Institute epidemiological database which documented co-morbidities through self-reporting.^8^ In an Indonesia based study, the authors identified a potential limitation as co-morbidities being largely self-reported or under-diagnosed.^9^ Overall testing rates may have been particularly low during the first phase of the pandemic when health systems were overwhelmed. From the studies reporting inpatient comorbidity rates, it is reasonable to assume self-reporting was the norm, limiting diagnosis of new cases.

To estimate the proportion of COVID-19 hospitalised cases which had UD in each country, we estimated the relative risk of hospitalisation based on the study from China^14^ discussed above, which categorised patients as having previously undiagnosed and diagnosed diabetes. This study grouped patients into 1) non-severe and 2) severe and critical patients as per Chinese COVID-19 management guidelines (version 6.0) — we used the prevalence of the latter where severe and critical cases represented 75% of all hospitalisations in this study. Combining the underlying adult population prevalence of diabetes^27^ with the proportion of COVID-19 hospitalised patients with previously undiagnosed and diagnosed diabetes in China, we estimated a relative risk of 1.09 for COVID-19 hospitalisation of undiagnosed vs. diagnosed diabetes patients.

Then the number of patients with UD by country was estimated as follows:

$${Hos}_{UD,c}^{0}={{POP}_{c}*RR}_{DD}^{UD}*{hr}_{DD,c}*p_{D,c}*p_{UD,c}$$

Where ${Hos}_{UD,c}^{0}$ is the population of COVID-19 hospitalised who are undiagnosed with diabetes, ${RR}_{DD}^{UD}$ is the relative risk (RR) of COVID-19 hospitalisation for those with undiagnosed relative to those with DD.

The number of COVID-19 hospitalised patients under an ‘intervention’ scenario where all previously UD patients are diagnosed was calculated as follows:

$${Hos}_{DD,c}^{1}={Hos}_{DD,c}^{0}\;+\;{hr}_{DD,c}*p_{D,c}*p_{UD,c}*{POP}_{c}$$

Where ${Hos}_{DD,c}^{1}$ is the number of COVID-19 hospitalised patients with DD at time 1 (after the intervention) in country *c*.

#### Deaths and QALYs

COVID-19 deaths in DD patients was estimated as follows:

$${Deaths}_{DD}={Hos}_{DD,c}^{0}*{dr}_{hos,DD,R}$$

Where ${dr}_{hos,DD,R}$ is the death rate for those hospitalised for COVID-19 with DD in region *R* and ${Hos}_{DD,c}^{0}$ is the total number of COVID-19 hospitalised individuals with DD. To estimate the death rate for DD patients we note that a December 2020 meta-analysis^5^ found that 24% of all COVID-19 hospitalised patients which had diabetes died. We adjusted this death rate similar to the adjustment for the overall CFR discussed in the previous section — therefore, a 31% and 9% reduced CFR in diabetes patients in African and Latin American countries, respectively.

Then COVID deaths for individuals with UD was calculated as follows:

$${Deaths}_{UD,c}={dr}_{hos,DD,R}*{{RR}_{DD}^{UD}}_{death}*{Hos}_{UD,c}^{0}$$

Where ${{RR}_{DD}^{UD}}_{death}$ is the relative risk of death of those with UD relative to those with DD and ${Hos}_{UD,c}^{0}$ as the total number of hospitalised individuals with undiagnosed disease.

The number of deaths in the intervention scenario was calculated as follows:

$${Deaths}_{DD,c}^{1}+{Deaths}_{UD,c}^{1}={Deaths}_{DD,c}^{0}+\;{dr}_{hos,DD,R}*{Hos}_{UD,c}^{0}$$

Then the change in quality-adjusted life years was calculated as follows:

$$△{QALYs}_{c}=△{Deaths}_{c}*{YLL}_{DD,c}*\left(1-\left(P_{CD}*{DW}_{CD}+\left({1-P}_{UND}\right)*{DW}_{UND}\right)\right)$$

Where ${YLL}_{DD,c}$ are the years of life lost for an individual with DD, $P_{CD}$ and $P_{UND}$ are the proportions of diabetes patients with complicated and uncomplicated diabetes respectively, and ${DW}_{CD}$ and ${DW}_{UND}$ are the disability weights for individuals with complicated and uncomplicated diabetes, respectively. The years of life gained are based on a study that estimated the average years of life lost per COVID death in 81 countries^21^ based on the average age of death and life expectancy within a country. We took the simple average of years of life lost for males and females. To take into account that YLL for individuals with diabetes would be lower due to lower life expectancy of diabetes patients we note that in the United Kingdom those with type 1 and type 2 diabetes had 20 years and 10 years lower life expectancy than those without diabetes.^28^^,^^29^ Using estimates of the proportion of the diabetes population with type 1 and type 2 diabetes globally,^30^^,^^31^ and UK life expectancy, we estimated that someone with diabetes will have 86% of the life expectancy of someone without diabetes. Using this factor, we estimated the average years of life lost for each COVID death of a diabetes patient in each country. We then weighted the years of life lost by the disability weights — 0.334 and 0.663 for diabetes patients with and without complications, respectively.^32^ The proportion of diabetes patients that develop complications was estimated at 63%.^33^ Key underlying demographic and epidemiological input data by country are provided in Table 1. Modelling parameter values and sources are presented in Table 3. The model was implemented in Microsoft Excel 2021 (with Visual Basic for Applications).

**Table 3:** Modelling parameters.

Parameter	Mean value	Source
Case fatality rate (hospitalised patients)	Adjusted for region using global mean of 0.20	Authors’ assumption based on Dorjee et al.,^5^ Clark et al.^17^
Case fatality rate (hospitalised diabetes patients)	Adjusted for region using global mean of 0.24	Authors’ assumption based on Dorjee et al.,^5^ Clark et al.^17^
Relative risk of hospitalization (newly diagnosed diabetes vs known diabetes)	1.09	Authors’ assumption based on Li et al.^14^
Relative risk of mortality, hospitalised patients (newly diagnosed diabetes vs known diabetes)	1.19	Authors’ assumption based on Li et al.^14^
Disability weight: Diabetes with complications	0.33	Ock et al.^32^
Disability weight: Diabetes without complications	0.66	Ock et al.^32^
% of diabetes population that develop complications	0.63	Boutayeb et al.^33^

#### Scenario and sensitivity analyses

To capture the uncertainty in the parameter values, we conducted one-way sensitivity analyses, varying the relative risk of hospitalisation and mortality for patients with previously UD. First, we changed the relative risk of hospitalisation for previously undiagnosed vs. diagnosed diabetes patients to 1.01 (instead of the base case value of 1.19). This is the estimated relative risk of all patients rather than only severe and critical cases which was used in the main analysis as discussed above.^14^ In a second and third model, we used two other values of the relative risk of mortality for previously undiagnosed vs. diagnosed diabetes patients (1.10 and 1.30, respectively). Additionally, uncertainty analysis was conducted using Monte Carlo simulations where all parameters, cases and deaths, were varied 20% above and below their mean value for every iteration, following previous modelling studies.^34^, ^35^, ^36^ The mean value of 1000 iterations and the 95% uncertainty interval are reported.

#### Ethics

This study used aggregate secondary data from published sources. There was no separate ethics clearance required.

#### Role of the funding source

This work was supported, in part, by the Bill & Melinda Gates Foundation (INV-029062) and FIND. The Bill & Melinda Gates Foundation had no role in study design, data collection and analysis, decision to publish, or preparation of the manuscript. Three co-authors are employees of FIND. All authors had full access to the data and accept responsibility for the decision to submit for publication.

## Results

The results are presented in Table 4 for hospitalisation related outcomes. We estimated that across the eight countries, 6.7 (95% uncertainty interval [UI]: 3.4–11.3) million COVID-19 hospitalised patients had UD. These represented 21.1% (95% CI: 13.4%–30.1%) of all COVID-19 hospitalisations. According to our modelled estimates, if these populations had been diagnosed and subsequently initiated on treatment at a similar rate to the general diabetes patient population prior to the pandemic, 1.7% (95% UI: −3.0% to 5.9%) of COVID-19 hospitalisations could have been prevented. Note there is a negative value of 95% lower bound of uncertainty range here and elsewhere as a results of the stochastic simulation process that incorporates potential scenarios in which the relative risk of undiagnosed diabetes vis-a-vis diagnosed diabetes is less than 1.

**Table 4:** Undiagnosed diabetes and COVID-19 hospitalizations.

Country	Hospitalizations				Scenario: All undiagnosed cases diagnosed prior to pandemic	
	Total	Diagnosed diabetes	Undiagnosed diabetes	Undiagnosed diabetes (% of total)	Decrease in Hospitalizations	Decrease in Hospitalizations (% of total)
Brazil	3,296,253	531,258	272,278	8.26	22,022	0.67
	[2,660,921–3,912,707]	[384,622–715,474]	[156,593–421,613]	[5.88–10.78]	[−29,532 to 85,435]	[−0.92 to 2.57]
China	86,195	6584	7858	9.12	637	0.74
	[69,581–102,314]	[4766–8866]	[4062–13,040]	[5.84–12.75]	[−903 to 2594]	[−1.05 to 3.0]
India	21,450,186	3,800,697	4,790,138	22.33	388,170	1.82
	[17,315,803–25,461,732]	[2,751,640–5,118,607]	[2,453,308–8,003,647]	[14.17–31.43]	[−548,316 to 1,585,959]	[−2.58 to 7.35]
Indonesia	935,371	115,172	407,818	43.60	33,285	3.58
	[755,085–1,110,302]	[83,383–155,109]	[152,069–943,402]	[20.14–84.97]	[−51,176 to 165,977]	[−5.65 to 17.59]
Mexico	1,871,551	616,391	617,979	33.02	50,044	2.68
	[1,510,822–2,221,562]	[446,257–830,128]	[328,967–1,010,589]	[21.77–45.49]	[−69,588 to 199,758]	[−3.78 to 10.64]
Nigeria	555,473	92,306	117,814	21.21	9547	1.73
	[448,409–659,356]	[66,828–124,314]	[60,203–197,365]	[13.43–29.93]	[−13,497 to 38,973]	[−2.45 to 6.99]
Pakistan	2,110,005	620,997	249,480	11.82	20,172	0.96
	[1,703,315–2,504,612]	[449,592–836,331]	[146,203–382,384]	[8.58–15.27]	[−26,999 to 78,749]	[−1.32 to 3.66]
South Africa	1,338,319	222,396	209,547	15.66	16,966	1.27
	[1,080,367–1,588,608]	[161,011–299,513]	[112,636–339,730]	[10.43–21.39]	[−23,431 to 67,396]	[−1.79 to 4.99]
Total	31,643,352	6,005,802	6,672,910	21.09	540,843	1.71
	[25,544,303–37,561,193]	[4,348,098–8,088,342]	[3,414,042–11,311,771]	[13.37–30.12]	[−763,441 to 2,224,841]	[−2.99 to 5.92]

Note: These estimates represent burden during the first stage of the pandemic — before a country reached a 1% population vaccination rate with one dose of a COVID-19 vaccine. 95% uncertainty interval provided in brackets.

**Fig. 1:**
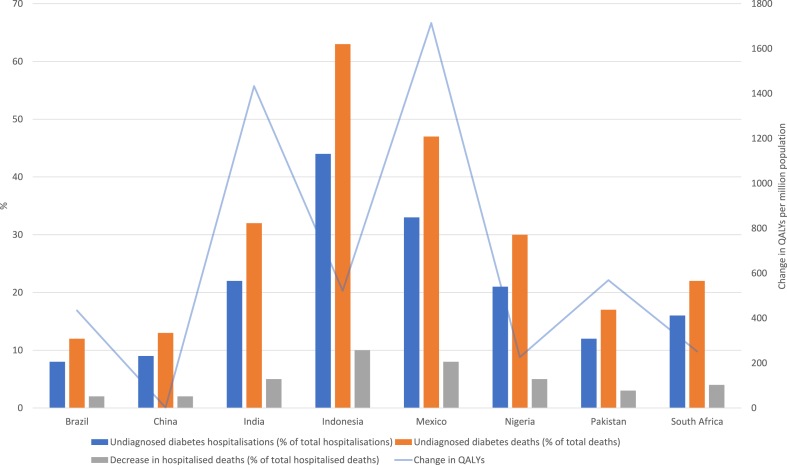
Key results summary, undiagnosed diabetes burden and changes in deaths and QALYs in intervention scenario. Note: These estimates represent burden during the first stage of the pandemic — before a country reached a 1% population vaccination rate with one dose of a COVID-19 vaccine. Undiagnosed diabetes deaths refer to deaths in hospitalised patients with undiagnosed diabetes. The decrease in hospitalised deaths is associated with the intervention. *QALY* = Quality-adjusted life year.

Table 5 presents the estimated UD mortality burden and Fig. 1 presents a visual summary of key results. We estimated that across the eight countries 1.9 (95% UI: 0.9–3.4) million COVID-19 deaths were of patients with UD, representing 30.5% (95% UI: 14.3%–55.5%) of all COVID-19 deaths. According to our model, If these populations had been diagnosed prior to the pandemic, 5.0% (95% UI: −0.9% to 14.1%) of COVID-19 deaths could have been prevented, and 1.8 (95% UI: −0.3 to 5.0) million QALYs could have been gained. The aggregate results in both Table 4, Table 5 are driven heavily by India given the country’s relatively large population size and COVID-19 burden — 73.7% of all decrease in deaths are from India in the intervention scenario.

**Table 5:** Undiagnosed diabetes and COVID-19 deaths.

Country	Deaths				Scenario: all undiagnosed cases diagnosed prior to pandemic		
	Total	Diagnosed diabetes	Undiagnosed diabetes	Undiagnosed diabetes (% of total)	Change in QALYs	Decrease in deaths	Decrease in deaths (% of total)
Brazil	599,150	115,999	71,090	11.87	67,317	11,642	1.94
	[395,181–880,842]	[76,473–170,712]	[36,845–119,284]	[6.15–19.91]	[−12,747 to 176,719]	[−2186 to 30,093]	[−0.36 to 5.02]
China	17,196	1578	2251	13.09	2131	368	2.14
	[11,145–24,853]	[1040–2322]	[1068–4014]	[6.21–23.34]	[−398 to 5989]	[−70 to 1025]	[−0.4 to 5.96]
India	4,279,321	910,837	1,372,310	32.07	1,298,841	224,537	5.25
	[2,824,565–6,290,143]	[600,477–1,340,447]	[645,102–2,464,852]	[15.07–57.60]	[−244,421 to 3,668,401]	[−42,460 to 630,392]	[−0.99 to 14.73]
Indonesia	186,607	27,601	116,777	62.58	110,402	19,068	10.22
	[122,635–273,804]	[18,196–40,619]	[40,703–283,835]	[21.81–152.10]	[−19,876 to 377,305]	[−3299 to 67,180]	[−1.77 to 36.0]
Mexico	340,186	134,588	161,320	47.42	152,708	26,403	7.76
	[224,098–499,092]	[88,728–198,068]	[78,638–280,275]	[23.12–82.39]	[−28,380 to 422,720]	[−4965 to 72,165]	[−1.46 to 21.21]
Nigeria	76,341	15,239	23,251	30.46	22,006	3804	4.98
	[50,948–111,801]	[10,046–22,427]	[10,906–41,718]	[14.29–54.65]	[−4150 to 62,225]	[−720 to 10,704]	[−0.94 to 14.02]
Pakistan	420,947	148,822	71,496	16.98	67,706	11,710	2.78
	[277,804–617,011]	[98,112–219,016]	[37,736–119,584]	[8.96–28.41]	[−12,800 to 175,804]	[−2211 to 30,098]	[−0.53 to 7.15]
South Africa	183,930	36,716	41,360	22.49	39,154	6770	3.68
	[120,532–269,092]	[24,205–54,033]	[20,334–71,714]	[11.06–38.99]	[−7297 to 107,734]	[−1271 to 18,465]	[−0.69 to 10.04]
Total	6,103,678	1,391,379	1,859,856	30.47	1,760,265	304,301	4.99
	[4,085,194–8,971,801]	[917,278–2,047,644]	[871,330–3,385,276]	[14.28–55.46]	[−330,068 to 4,996,897]	[−57,181 to 860,121]	[−0.94 to 14.09]

Note: These estimates represent burden during the first stage of the pandemic — before a country reached a 1% population vaccination rate with one dose of a COVID-19 vaccine. 95% uncertainty interval provided in brackets. *QALY* = Quality-adjusted life year.

Indonesia, followed by Mexico and Nigeria, had the highest proportion of total estimated COVID-19 hospitalisations and deaths with UD, while Brazil had the lowest. For Indonesia, these results are driven by the highest proportion of UD amongst all LMICs analysed, where 74% of diabetes patients are undiagnosed.^4^ For Brazil, the estimates are driven by the relatively low prevalence of diabetes in COVID-19 hospitalised patients and relatively low level of UD — 32% of all diabetes cases,^4^ which is the second lowest UD prevalence of the studied countries. In per capita terms, Fig. 1 shows that the greatest QALYs could have been gained in Mexico, followed by India, Pakistan and India, if these patients had been diagnosed prior to the pandemic. In these countries, in addition to overall diabetes burden, these results are driven by the higher number of overall COVID-19 deaths and also the proportion of these deaths which are comprised of patients with DD.

Appendix Tables A1–A4 present the results of the sensitivity analyses. Appendix Table A1 shows the results using a relative risk of hospitalisation for undiagnosed diabetes vs. previously diagnosed diabetes of 1.01 instead of the base case scenario of 1.09. In this scenario, 19.6% (95% UI: 12.6%–28.6%) of all COVID-19 hospitalisations had UD, and a prior diagnosis of these cases would have decreased 0.2% (95% CI: −5.0% to 4.1%) of COVID-19 hospitalisations. Appendix Tables A3 and A4 show results for low and high mortality scenarios for UD patients, respectively. In the low mortality scenario, 28.2% (95% UI: 13.2%–51.3%) of COVID-19 deaths were of patients with UD; 1.0 (95% UI: 1.1–3.6) million QALYs could have been gained if these diabetes cases had been diagnosed prior to the pandemic. In the high mortality scenario, 33.3% (95% UI: 15.6%–60.6%) of COVID-19 deaths were of patients with UD; 2.8 (95% UI: 0.4–6.7) million QALYs could have been gained if these diabetes cases had been diagnosed prior to the pandemic.

## Discussion

The burden of NCDs is expected to increase globally, especially in LMICs. Diabetes caused 59.2 million DALYs in LMICs in 2019.^1^ This growing burden is due to an increased preference towards unhealthy diets and sedentary lifestyles, but also low levels of diagnostic and treatment capacity. There are many diabetes prevention interventions that are highly cost-effective in low-income and middle-income settings; however, when cases cannot be prevented, appropriate diagnostics, treatment, and control are vital. Our modelling estimates suggested that across the eight countries, 6.7 million COVID-19 hospitalised patients had undiagnosed diabetes of which 1.9 million died. These represented 21.1% of all COVID-19 hospitalisations and 30.5% of all COVID-19 deaths in these countries. We find that if these populations had been diagnosed for diabetes prior to the COVID-19 pandemic, 1.7% of COVID-19 hospitalisations and 5.0% of COVID-19 deaths could have been prevented, and 1.8 million QALYs gained.

The annual burden of disease from diabetes and other NCDs — in terms of morbidity and mortality — has significantly increased during COVID-19. This burden is particularly difficult to estimate, when a large proportion of underlying NCDs are undiagnosed. While future variants of COVID-19, their severity, and association to different NCD risk factors are uncertain at this time, scenario analysis may demonstrate the benefits of preventing additional NCDs and diagnosing current undetected cases. Besides COVID-19, other future infectious disease outbreaks may also create a case for investing in prevention and diagnostics. Past epidemic diseases including the Ebola virus, severe acute respiratory syndrome (SARS), and Middle East respiratory syndrome (MERS), have been more severe in populations with NCDs, and it is likely they would be for future infectious diseases as well.

Our findings have important implications for clinical practise and future research in this area. At present, an estimated 45% of individuals with diabetes globally are not aware of their disease status.^4^ A 2016 systematic review found that type 2 diabetes screening was cost-effective in 62% of studies.^37^ In studies comparing scenarios, targeted or opportunistic screening was more cost-effective than universal screening.^37^ However, studies in this review were largely concentrated in higher-income countries. Future research should incorporate scenarios where outbreaks of novel diseases such as COVID-19 may severely increase both the health and financial costs of having undiagnosed diabetes, making universal screenings potentially cost-effective vis-à-vis targeted screenings. Based on current available evidence, our findings make a case for increased testing of diabetes among at-risk populations.

Studies have shown that the relationship between COVID-19 and diabetes may be bidirectional.^12^ A recent study found there is an increased risk of diabetes 12 months after COVID-19 infection.^38^ Therefore, COVID-19 may exacerbate the present burden of NCDs and necessitate even more emphasis on testing and management of diabetes. Future policy can consider increased NCD monitoring and testing in COVID-19 infected individuals during and post-care.

There are several important limitations to our analysis. First, there is uncertainty regarding some of the parameters and data used in this study. Quality of data on number of cases and deaths are influenced by local surveillance capacities. Some parameters on prevalence of disease in hospitalised patients come from single centre studies within countries but are consistent with estimates from cross-country meta-analyses. However, even meta-analyses relied heavily on surveillance studies from early epicentres of COVID such as China, or high-income countries. These are inherent limitations of all COVID-19 analyses. We have minimised these biases by using region-specific parameters whenever possible, from highly cited, published estimates and have conducted uncertainty analyses, varying factors that could have an undue influence on the findings of this study.

Second, we have relied on meta-analyses and studies to document if patients had previously diagnosed diabetes, previously undiagnosed diabetes, or were new diabetes patients. If a proportion of the patients with diagnosed diabetes were actually patients with previously undiagnosed diabetes, but were not explicitly documented, our estimates of the undiagnosed diabetes burden could be overestimated. However, several studies suggest lack of new diagnosis and reliance on self-reporting of co-morbidities in health centres.^7^, ^8^, ^9^ Third, our analysis is also limited by the availability of data on previously undiagnosed diabetes in COVID-19 hospitalised patients. We used parameters from a study from China that provided mortality outcomes for those with previously undiagnosed diabetes vs. those with known diabetes. To capture regional variations in mortality, we conducted sensitivity analyses by considering alternative values of the relative risk of mortality for those with previously undiagnosed diabetes. Lastly, due to lack of data, we were unable to incorporate certain characteristics of the pandemic into our modelling such as COVID strains and severity. The focus on the first phase of the pandemic — defined as prior to achieving a 1% vaccination rate in our model — however, limited the number of strains circulating in the population.

Non-communicable diseases, specifically diabetes, obesity, and hypertension, are rapidly growing in LMICs, afflicting a serious health and economic burden. Their importance has been magnified during the COVID-19 pandemic. This study highlights the benefits of prevention of diabetes cases, and also the importance of timely diagnosis to reduce future disease burdens. Future research can extend our modelling to other phases of the pandemic to account for the full burden of disease caused by undiagnosed NCDs, use updated parameters as they become available, or incorporate other indirect costs such as health burden caused by reduced availability of resources for other patients, health expenditure and caregiver costs, and reduced productivity.

## Contributors

AS and RL designed the study. AS conducted the analysis and wrote the first version of the manuscript. All authors had full access to the data and AS and AN take responsibility for the integrity of the findings. AS and AN have verified the underlying data for the analysis. All authors critically evaluated the methodology and results, and reviewed and edited the manuscript. All authors accepted the responsibility to submit for publication.

## Data sharing statement

All data are available upon request to the corresponding author.

## Declaration of interests

Co-authors SC, SO, and BV are employees of FIND.

## Appendix ASupplementary data

Appendix Tables A1–A4
