# Supplementary material for: Estimates of hospitalisations and deaths in patients with COVID-19 associated with undiagnosed diabetes during the first phase of the pandemic in eight low-income and middle-income countries: a modelling study
Source: eClinicalMedicine. 2024 Mar 5;70:102492. doi: 10.1016/j.eclinm.2024.102492 (PMC10933463; doi:10.1016/j.eclinm.2024.102492)
Supplement: Appendix Tables A1–A4 [file mmc1.pdf]

Table A1: Undiagnosed diabetes and COVID-19 hospitalizations, low undiagnosed diabetes hospitalization scenario

Country	Hospitalizations				Scenario: All undiagnosed cases diagnosed prior to pandemic	
	Total	Diagnosed diabetes	Undiagnosed diabetes	Undiagnosed diabetes (% of total)	Decrease in Hospitalizations	Decrease in Hospitalizations (% of total)
Brazil	3,296,253	531,258	252,354	7.66	2,098	0.06
	[2,660,921 to 3,912,707]	[384,622 to 715,474]	[146,278 to 398,246]	[5.50 to 10.18]	[-50,451 to 58,518]	[-1.56 to 1.73]
China	86,195	6,584	7,285	8.45	64	0.07
	[69,581 to 102,314]	[4,766 to 8,866]	[3,833 to 12,387]	[5.51 to 12.11]	[-1,495 to 1,811]	[-1.79 to 2.03]
India	21,450,186	3,800,697	4,440,862	20.70	38,894	0.18
	[17,315,803 to 25,461,732]	[2,751,640 to 5,118,607]	[2,312,989 to 7,581,931]	[13.36 to 29.78]	[-918,733 to 1,107,574]	[-4.41 to 5.01]
Indonesia	935,371	115,172	378,342	40.45	3,810	0.4
	[755,085 to 1,110,302]	[83,383 to 155,109]	[141,637 to 893,175]	[18.76 to 80.44]	[-91,155 to 112,628]	[-9.76 to 12.42]
Mexico	1,871,551	616,391	572,864	30.61	4,929	0.26
	[1,510,822 to 2,221,562]	[446,257 to 830,128]	[309,706 to 960,775]	[20.50 to 43.25]	[-115,267 to 138,506]	[-6.44 to 7.25]
Nigeria	555,473	92,306	109,224	19.66	958	0.17
	[448,409 to 659,356]	[66,828 to 124,314]	[56,749 to 186,851]	[12.66 to 28.34]	[-22,639 to 27,234]	[-4.19 to 4.77]
Pakistan	2,110,005	620,997	231,215	10.96	1,907	0.09
	[1,703,315 to 2,504,612]	[449,592 to 836,331]	[135,569 to 358,692]	[7.96 to 14.32]	[-46,190 to 53,497]	[-2.22 to 2.45]
South Africa	1,338,319	222,396	194,245	14.51	1,664	0.12
	[1,080,367 to 1,588,608]	[161,011 to 299,513]	[106,084 to 324,537]	[9.82 to 20.43]	[-39,084 to 46,821]	[-3.04 to 3.42]
Total	31,643,352	6,005,802	6,186,391	19.55	54,323	0.17
	[25,544,303 to 37,561,193]	[4,348,098 to 8,088,342]	[3,212,845 to 10,716,594]	[12.58 to 28.53]	[-1,285,014 to 1,546,589]	[-5.03 to 4.12]

Note: These estimates represent burden during the first stage of the pandemic — before a country reached a 1% population vaccination rate with one dose of a COVID-19 vaccine. 95% uncertainty interval provided in brackets.
Source: Authors' estimates.

Table A2: Undiagnosed diabetes and COVID-19 deaths, low undiagnosed diabetes hospitalization scenario

Country	Deaths				Scenario: All undiagnosed cases diagnosed prior to pandemic		
	Total	Diagnosed diabetes	Undiagnosed diabetes	Undiagnosed diabetes (% of total)	Change in QALYs	Decrease in deaths	Decrease in deaths (% of total)
Brazil	599,150	115,999	65,903	11.00	62,574	10,821	1.81
	[599,150 to 599,150]	[76,473 to 170,712]	[34,237 to 110,066]	[5.71 to 18.37]	[-11,354 to 166,893]	[-1,844 to 28,502]	[-0.31 to 4.76]
China	17,196	1,578	2,088	12.14	1,981	342	1.99
	[17,196 to 17,196]	[1,040 to 2,322]	[986 to 3,698]	[5.73 to 21.51]	[-343 to 5,580]	[-60 to 956]	[-0.35 to 5.56]
India	4,279,321	910,837	1,272,575	29.74	1,207,373	208,729	4.88
	[4,279,321 to 4,279,321]	[600,477 to 1,340,447]	[595,421 to 2,272,831]	[13.91 to 53.11]	[-208,398 to 3,417,438]	[-36,736 to 586,430]	[-0.86 to 13.7]
Indonesia	186,607	27,601	108,387	58.08	102,663	17,732	9.5
	[186,607 to 186,607]	[18,196 to 40,619]	[37,056 to 263,268]	[19.86 to 141.08]	[-17,072 to 364,345]	[-3,034 to 63,070]	[-1.63 to 33.8]
Mexico	340,186	134,588	149,580	43.97	141,952	24,543	7.21
	[340,186 to 340,186]	[88,728 to 198,068]	[72,620 to 259,186]	[21.35 to 76.19]	[-24,943 to 398,704]	[-4,264 to 67,349]	[-1.25 to 19.8]
Nigeria	76,341	15,239	21,562	28.24	20,457	3,536	4.63
	[76,341 to 76,341]	[10,046 to 22,427]	[10,065 to 38,533]	[13.18 to 50.47]	[-3,527 to 58,040]	[-623 to 9,950]	[-0.82 to 13.03]
Pakistan	420,947	148,822	66,275	15.74	62,936	10,885	2.59
	[420,947 to 420,947]	[98,112 to 219,016]	[34,720 to 109,533]	[8.25 to 26.02]	[-11,415 to 166,524]	[-1,860 to 28,771]	[-0.44 to 6.83]
South Africa	183,930	36,716	38,349	20.85	36,396	6,293	3.42
	[183,930 to 183,930]	[24,205 to 54,033]	[18,763 to 66,430]	[10.20 to 36.12]	[-6,408 to 101,348]	[-1,090 to 17,171]	[-0.59 to 9.34]
Total	6,103,678	1,391,379	1,724,719	28.26	1,636,331	282,882	4.63
	[6,103,678 to 6,103,678]	[917,278 to 2,047,644]	[803,868 to 3,123,545]	[13.17 to 51.17]	[-283,461 to 4,678,873]	[-49,511 to 802,199]	[-0.81 to 13.14]

Note: These estimates represent burden during the first stage of the pandemic — before a country reached a 1% population vaccination rate with one dose of a COVID-19 vaccine.. 95% uncertainty interval provided in brackets. *QALY*= Quality-adjusted life year
Source: Authors' estimates.

Table A3: Undiagnosed diabetes and COVID-19 deaths, low undiagnosed diabetes mortality scenario

Country	Deaths				Scenario: All undiagnosed cases diagnosed prior to pandemic		
	Total	Diagnosed diabetes	Undiagnosed diabetes	Undiagnosed diabetes (% of total)	Change in QALYs	Decrease in deaths	Decrease in deaths (% of total)
Brazil	599,150	115,999	65,726	10.97	36,343	6,278	1.05
	[395,181 to 880,842]	[76,473 to 170,712]	[34,065 to 110,284]	[5.69 to 18.41]	[-40,708 to 128,566]	[-6,894 to 21,630]	[-1.15 to 3.61]
China	17,196	1,578	2,081	12.10	1,150	199	1.15
	[11,145 to 24,853]	[1,040 to 2,322]	[988 to 3,711]	[5.75 to 21.58]	[-1,316 to 4,366]	[-219 to 738]	[-1.27 to 4.29]
India	4,279,321	910,837	1,268,768	29.65	700,743	120,995	2.83
	[2,824,565 to 6,290,143]	[600,477 to 1,340,447]	[596,428 to 2,278,877]	[13.94 to 53.25]	[-797,870 to 2,673,659]	[-134,640 to 453,849]	[-3.15 to 10.61]
Indonesia	186,607	27,601	107,966	57.86	59,461	10,257	5.5
	[122,635 to 273,804]	[18,196 to 40,619]	[37,632 to 262,420]	[20.17 to 140.63]	[-71,767 to 272,018]	[-12,632 to 49,456]	[-6.77 to 26.5]
Mexico	340,186	134,588	149,149	43.84	82,407	14,231	4.18
	[224,098 to 499,092]	[88,728 to 198,068]	[72,704 to 259,128]	[21.37 to 76.17]	[-94,977 to 304,467]	[-15,485 to 51,508]	[-4.55 to 15.14]
Nigeria	76,341	15,239	21,497	28.16	11,873	2,050	2.69
	[50,948 to 111,801]	[10,046 to 22,427]	[10,083 to 38,570]	[13.21 to 50.52]	[-13,501 to 45,350]	[-2,286 to 7,706]	[-2.99 to 10.09]
Pakistan	420,947	148,822	66,101	15.70	36,557	6,315	1.5
	[277,804 to 617,011]	[98,112 to 219,016]	[34,889 to 110,561]	[8.29 to 26.26]	[-40,693 to 128,497]	[-6,985 to 21,629]	[-1.66 to 5.14]
South Africa	183,930	36,716	38,240	20.79	21,130	3,649	1.98
	[120,532 to 269,092]	[24,205 to 54,033]	[18,800 to 66,303]	[10.22 to 36.05]	[-24,303 to 77,377]	[-3,987 to 13,097]	[-2.17 to 7.12]
Total	6,103,678	1,391,379	1,719,528	28.17	949,663	163,973	2.69
	[4,085,194 to 8,971,801]	[917,278 to 2,047,644]	[805,587 to 3,129,854]	[13.20 to 51.28]	[-1,085,136 to 3,634,300]	[-183,127 to 619,613]	[-3.0 to 10.15]

Note: These estimates represent burden during the first stage of the pandemic — before a country reached a 1% population vaccination rate with one dose of a COVID-19 vaccine. 95% uncertainty interval provided in brackets. *QALY*= Quality-adjusted life year
Source: Authors' estimates.

Table A4: Undiagnosed diabetes and COVID-19 deaths, high undiagnosed diabetes mortality scenario

Country	Deaths				Scenario: All undiagnosed cases diagnosed prior to pandemic		
	Total	Diagnosed diabetes	Undiagnosed diabetes	Undiagnosed diabetes (% of total)	Change in QALYs	Decrease in deaths	Decrease in deaths (% of total)
Brazil	599,150	115,999	77,677	12.96	105,351	18,228	3.04
	[395,181 to 880,842]	[76,473 to 170,712]	[40,258 to 130,336]	[6.72 to 21.75]	[16,626 to 236,692]	[2,794 to 40,995]	[0.47 to 6.84]
China	17,196	1,578	2,460	14.30	3,335	577	3.36
	[11,145 to 24,853]	[1,040 to 2,322]	[1,167 to 4,386]	[6.79 to 25.51]	[508 to 8,006]	[91 to 1,379]	[0.53 to 8.02]
India	4,279,321	910,837	1,499,453	35.04	2,033,271	351,680	8.22
	[2,824,565 to 6,290,143]	[600,477 to 1,340,447]	[704,870 to 2,693,218]	[16.47 to 62.94]	[308,340 to 4,912,798]	[55,946 to 847,176]	[1.31 to 19.8]
Indonesia	186,607	27,601	127,596	68.38	172,955	29,887	16.02
	[122,635 to 273,804]	[18,196 to 40,619]	[44,474 to 310,132]	[23.83 to 166.20]	[21,231 to 516,648]	[3,487 to 89,237]	[1.87 to 47.82]
Mexico	340,186	134,588	176,267	51.81	239,034	41,349	12.15
	[224,098 to 499,092]	[88,728 to 198,068]	[85,923 to 306,242]	[25.26 to 90.02]	[36,924 to 563,166]	[6,493 to 97,500]	[1.91 to 28.66]
Nigeria	76,341	15,239	25,405	33.28	34,450	5,959	7.81
	[50,948 to 111,801]	[10,046 to 22,427]	[11,916 to 45,583]	[15.61 to 59.71]	[5,218 to 83,431]	[947 to 14,385]	[1.24 to 18.84]
Pakistan	420,947	148,822	78,120	18.56	105,955	18,334	4.36
	[277,804 to 617,011]	[98,112 to 219,016]	[41,232 to 130,663]	[9.80 to 31.04]	[16,571 to 237,842]	[2,787 to 40,802]	[0.66 to 9.69]
South Africa	183,930	36,716	45,192	24.57	61,286	10,602	5.76
	[120,532 to 269,092]	[24,205 to 54,033]	[22,218 to 78,358]	[12.08 to 42.60]	[9,507 to 143,918]	[1,660 to 24,928]	[0.9 to 13.55]
Total	6,103,678	1,391,379	2,032,170	33.29	2,755,637	476,615	7.81
	[4,085,194 to 8,971,801]	[917,278 to 2,047,644]	[952,058 to 3,698,918]	[15.60 to 60.60]	[414,925 to 6,702,502]	[74,206 to 1,156,402]	[1.22 to 18.95]

Note: These estimates represent burden during the first stage of the pandemic — before a country reached a 1% population vaccination rate with one dose of a COVID-19 vaccine. 95% uncertainty interval provided in brackets. *QALY*= Quality-adjusted life year
Source: Authors' estimates.
